# Cardiac rehabilitation via telerehabilitation in COVID-19 pandemic situation

**DOI:** 10.1186/s43044-021-00156-7

**Published:** 2021-03-29

**Authors:** Dian M. Sari, Laurentia C. G. Wijaya

**Affiliations:** 1 Department of Physical Medicine and Rehabilitation, Faculty of Medicine, Universitas Padjadjaran, Bandung, Indonesia; 2 Faculty of Medicine, Maranatha Christian University, Bandung, Indonesia

**Keywords:** Cardiac rehabilitation, Coronavirus disease-2019 pandemic, Home-based exercise, Telerehabilitation

## Abstract

**Background:**

Adherence to medication and lifestyle changes are very important in the secondary prevention of cardiovascular disease. One of the ways is by doing a cardiac rehabilitation program.

**Main body of the abstract:**

Cardiac rehabilitation program is divided into three phases. The cardiac rehabilitation program’s implementation, especially the second phase, center-based cardiac rehabilitation (CBCR), has many barriers not to participate optimally. Therefore, the third phase, known as home-based cardiac rehabilitation (HBCR), can become a substitute or addition to CBCR. On the other hand, this phase is also an essential part of the patients’ functional capacity. During the coronavirus disease-2019 pandemic, HBCR has become the leading solution in the cardiac rehabilitation program’s sustainability. Innovation is needed in its implementation, such as telerehabilitation. So, the cardiac rehabilitation program can be implemented by patients and monitored by health care providers continuously.

**Short conclusion:**

Physicians play an essential role in motivating patients and encouraging their family members to commit to a sustainable CR program with telerehabilitation to facilitate its implementation.

## Background: cardiac rehabilitation

Cardiac rehabilitation (CR) is a comprehensive intervention for secondary prevention of cardiovascular disease (CVD) [1–3]. The 2016 European guidelines on CVD prevention in clinical practice emphasize that medication adherence and lifestyle changes are essential in secondary preventions of CVD, which can be increased and improved through CR programs; therefore, reducing the incidence of recurrent heart disease and the risk of death [2]. This program’s ultimate goal is to prevent cardiac-related disease recurrence, lower the risk of other cardiac events, such as arrhythmias or heart failure (HF), and improve patients’ mental health status and quality of life cardiac-related diseases [3].

The cardiac rehabilitation program focuses on risk assessment and management [4]. This program implements a preventive lifestyle to control risk factors of cardiac diseases, such as obesity, hypertension, diabetes, and dyslipidemia. Cardiac rehabilitation includes medical evaluation; physical exercise, controlling nutritional intake, lipid levels, and blood pressure (BP); planning programs to reduce cigarette smoking and alcohol consumption; stress management; modified individualized lifestyle consultation; tailored targeted pharmacological therapy; patient education; and psychological counseling [1, 3]. The CR program components are aerobic training, strength/resistance exercise, flexibility, posture, coordination, and balance [5–7]. Table 1 below simplifies the predictors to assess high-risk patients during cardiac rehabilitation [8].

**Table 1:** Patients with high risk during cardiac rehabilitation [8]

Ischemic risk	Arrhythmia risk
Postoperative angina	Acute infarction within 6 weeks
Left ventricular ejection fraction (LVEF) <35%	Active ischemia by angina or exercise testing
NYHA grade III or IV congestive heart failure (CHF)	Significant left ventricular dysfunction (LVEF <30%)
Ventricular tachycardia or fibrillation in the postoperative period	History of sustained ventricular tachycardia
The systolic blood pressure drop of 10 mmHg or more with exercise	History of sustained life-threatening supraventricular arrhythmia
Incapable of self-monitoring	History of sudden death
Myocardial ischemia with exercise	Initial therapy of patients with automatic implantable cardioverter-defibrillator (AICD)
	Initial therapy of a patient with a rate-adaptive cardiac pacemaker

This literature review aimed to promote telemedicine specifically in CR through telerehabilitation programs among physicians and encourage each family member to actively support the continuum of rehabilitation programs at home to maintain and improve patients’ quality of life.

## Main text: phases of cardiac rehabilitation

Cardiac rehabilitation is currently divided into three phases. The first phase is the acute phase, which starts from hospitalization following the heart attack up to the discharge, includes early mobilization programs. The second phase is the supervised rehabilitation phase initiated after complete healing and characterized by intense education and aerobic activities to achieve the desired exercise results. The third phase is the phase devoted to maintaining aerobic activities in phase II through a regular exercise program [8].

### Phase I (acute phase)

The acute phase includes patient assessment, early mobilization, identification of CVD risk factors, and pre-discharge assessment. In this phase, outpatients are prepared to enter CR. Patients are given primary education on how to control risk factors and do self-care. The CR program is designed to make individuals mobilize from bed until they climb two stairs within 3–5 days. The types of activities commonly used in early mobilization are shown in Table 2 [8, 9].

**Table 2:** Types of activities commonly used in early cardiac rehabilitation [9]

Types of activities	Methods	METs
Toileting	Bedpan	1.5–2.5
	Commode	
	Urinal (in bed)	
	Urinal (standing)	
Bathing	In bed	1.5–2.0
	In bathtub	
	Shower	
Walking	Flat surface	
	2 m/h	2.0–2.5
	2.5 m/h	2.5–2.9
	3 m/h	3.0–3.3
Upper body exercise	While standing	
	Arms movement	2.5–3.0
	Trunk movement	
Stairs climbing	One flight = 12 steps	
	Down one flight	3.0–4.0
	One to two flights	

*METs* metabolic equivalent tasks

Phase I’s primary goal is that patients are capable of performing activities up to 4 metabolic equivalent tasks (METs). This value represents the estimated number of daily activities at home after discharge. Before discharge, patients perform an exercise test to determine their functional capacity, and then they are educated about which activities can be performed at home [8].

### Phase II

The second phase begins as soon as patients are allowed to be discharged. The phase II or center-based cardiac rehabilitation (CBCR) phase is a program that provides resources and an environment, i.e., a supportive community for patients to complete their comprehensive rehabilitation program [10]. The CR program generally consists of 3 sessions per week for ± 8 weeks. Patient risk stratification is necessary to determine the exercise prescription. The criteria of high-risk patients in CR programs are listed in Table 3 [7–9].

**Table 3:** Characteristics of patients based on risk stratification [7, 9]

**Low risk for exercise participation**	
• Absence of complex ventricular dysrhythmia during exercise testing and recovery	
• Absence of angina or other significant symptoms	
• Presence of normal hemodynamics during exercise testing and recovery • Functional capacity ≥ 7 METs	
Non-exercise testing findings	
• Rest ejection fraction ≥ 50% • Uncomplicated myocardial infarction or revascularization procedure	
• Absence of CHF	
• Absence of signs or symptoms of post-event or post-procedure ischemia	
• Absence of clinical depression	
**Moderate risk for exercise participation**	
• Presence of complex ventricular arrhythmias during exercise testing or recovery	
• Mild to moderate level of ischemia during exercise testing or recovery	
• Functional capacity < 5 METs	
Non-exercise testing findings	
• Rest ejection fraction 40–49%	
**High risk for exercise participation**	
• Presence of complex ventricular arrhythmias during exercise testing or recovery	
• Presence of angina or other significant symptoms	
• High level of ischemia during exercise testing or recovery	
• Presence of abnormal hemodynamics with exercise testing	
Non-exercise testing findings	
• Rest ejection fraction <40%	
• History of cardiac arrest or sudden death	
• Complex dysrhythmias at rest	
• Complicated myocardial infarction or revascularization procedure	
• Presence of CHF	
• Presence of signs or symptoms of post-event or post-procedure ischemia	
• Presence of clinical depression	

*METs* metabolic equivalent tasks

*CHF* congestive heart failure

In every risk category, direct supervision should be conducted. The supervision includes electrocardiogram (ECG) and hemodynamic examination. In this phase, a patient will undergo lung and neuromusculoskeletal examination, resting ECG, and risk profile to get a treadmill test [11].

### Phase III (maintenance phase)

The third phase is the essential part of the program yet usually receives the least attention. The benefits gained from phase II (CBCR) will be subsided within a few weeks if they stop exercising [8]. Therefore, home-based cardiac rehabilitation (HBCR) may overcome this problem and be used as an adjunct or alternative to CBCR [10].

From the beginning of the CR program, sustaining an exercise program needs to be seriously emphasized. The exercises need to be integrated into patients’ actual lifestyles to facilitate compliance in this phase. Moderate-intensity exercises should be performed at the heart rate target for at least 30 min, three times/week, while low-intensity exercises should be performed five times/week [8].

If peak heart rate (HR) is unknown, we can use Borg scale, either the Borg Rating of Perceived Exertion (RPE) scale (primarily the Borg 6-20 RPE scale/BORG-RPE) or the Borg Category-Ratio-10 scale (BORG-CR10) to guide exercise intensity. For moderate intensity which scores 12–13 on BORG RPE (4–6 on BORG-CR10), is in somewhat hard criteria or having a target as 40–59% of HR reserve, while low intensity which scores below 12 on BORG RPE (<3 on BORG-CR10) is in light criteria or having target as <40% of HR reserve. Heart rate reserve is the difference between a person’s measured heart rate (predicted maximum heart rate) and the resting heart rate [7]. ECG monitoring is not necessary during this phase [8].

## Benefits of cardiac rehabilitation

Cardiac rehabilitation post-myocardial infarction (MI), post coronary artery bypass graft (CABG), and post percutaneous coronary intervention (PCI) are recommended (Class IA) by the American Heart Association (AHA) [12] and European Society of Cardiology (ESC) guidelines [1, 13, 14]. Cardiac rehabilitation positively impacts cardiorespiratory endurance, muscle mass and strength, mobility, physical activity, social interaction, cognitive performance, mood, and vitality of patients [3, 15]. This program is proven to improve self-care in the elderly, avoid unnecessary hospitalizations, and improve patients’ quality of life [15]. Secondary prevention, including CR, is crucial to improve the long-term prognosis of patients with MI and increase their functional capacity [4]. Cardiac rehabilitation is also considered to improve cardiorespiratory fitness and heart rate recovery, which are indicators of autonomic function in coronary artery disease (CAD) patients with a previous MI history. Cardiac rehabilitation may reduce the length of stay, depression rate, the incidence of recurrent acute coronary syndrome, disability, stroke, and on the other hand, improve medication adherence [15–17].

The rehabilitation program benefits are gained from the direct physiological effects of physical exercises and the indirect effects of risk factors control, lifestyle modification, and mood. Several studies had shown that patients who underwent CR programs following primary PCI had a lower mortality rate [13, 16].

## Types of cardiac rehabilitation

The CR programs are available in two forms: center-based cardiac rehabilitation (CBCR) and home-based cardiac rehabilitation (HBCR). The CBCR exercises are conducted in hospitals or specialized institutions, which is safer for cardiac disease patients because professionals supervise them. However, the CBCR program cost is quite expensive since it is a long-term program and may not be convenient for patients living in rural areas [3]. Therefore, HBCR is recommended for patients who experience limitations to CBCR. Patients who dislike group activities but are disciplined and have leisure time are the ideal candidates for HBCR [10]. In the HBCR program, patients can still participate and perform exercises independently at home or any local fitness center [3].

The CBCR program had a very high dropout rate (up to 24–50%), suggesting that the long-term exercises of CBCR were not continuously performed by most of the patients, whereas the progress been made after completing the program will not be maintained if the exercises are stopped [3, 18]. Moreover, lower exercise compliance caused increased body weight, and lipid levels were reported as patients stopped the CBCR program. Therefore, these exercises are only recommended at the beginning of the rehabilitation program, and then patients are recommended to continue on the HBCR program [3].

Both CBCR and HBCR programs effectively improved clinical outcomes and health-related quality of life (HRQoL) in patients with CAD, MI, or low-risk HF. The efficacy of HBCR was comparable to CBCR in terms of increased aerobic capacity, level of physical activity, exercise compliance, controlled BP, and cholesterol level. The HBCR effectively reduced cardiac mortality in patients with CAD, ranging between 27 and 31% [5, 18, 19].

Patients frequently do not participate in CR due to their low perception of the program’s importance and necessity. The less optimal participation may lead to less optimal results in CR [3, 16].

The primary strategy for improving adherence in rehabilitation works through a strong doctor-patient relationship and good communication skills, ensuring patient understanding of the impacts of non-adherence. Physician support indeed influences patient participation in CR programs. Patient characteristics may be the significant predictors when referring patients to join CR programs [2, 10].

Of all patients who were referred to CR, only 30–34% participated [10]. The low rate of patient participation is influenced by several factors, including lack of physician referral or low level of physician support, low socioeconomic status, lack of education, racial/ethnic minority status, barriers in transportation, current routine activities at home, older age, female gender, lack of friends, and the presence of depression. The participation rate in CR is lower in women than men because women tend to do household chores, have low interest, and comorbidities such as knee and back pain [3, 10].

The HBCR program has proven to be safe, cost-effective, and acceptable to communities. In Indonesia, patients considered safe to do the HBCR program are commonly categorized as low-risk patients, do not require ECG monitoring, and can be monitored by daily reports via logbook or diary. Compared to CBCR, most patients can complete the HBCR program. Self-monitoring and self-management in HBCR will make a better transition from active interventions to lifelong independence of patients. The HBCR is considered safe because there are no reports or evidence of increased hospitalization or death risk. These findings support the HBCR program applied to HF patients as an alternative to conventional hospital-based rehabilitation [10, 18, 20].

The HBCR program includes the core components of the CBCR program, which is delivered by a health care professional but may be delivered through telephone calls or cellular technologies. This program may expand the modalities of patient education, counseling, and monitoring because this service, which has been implemented in the USA, can be accessed 24 h a day and 7 days a week; therefore, making it easier to be accessed compared to contact professionals in the CBCR [18].

The use of information and communication technology may be worth adjunctive to CR. It is known as telemedicine. Telemedicine is considered suitable, safe, and effective in increasing exercise capacity, controlling shortness of breath, maintaining physical activity, reducing disability, and improving quality of life in elderly patients with chronic obstructive pulmonary disease (COPD), CHF, and ischemic heart disease [1, 17, 18]. The virtual CR program is a part of HBCR innovations to facilitate or maximize care quality and effectiveness. This innovation works through communication via telephone and video conferencing, e-mails, letters, text messaging, smartphone applications, and online platforms. Telemedicine applied in CR, called telerehabilitation, may become a solution for a more optimal HBCR implementation. This program is targeted for low-to-moderate risk patients, whereas high-risk patients need to continue their rehabilitation through the CBCR program [17, 18, 21–23].

**Fig. 1:**
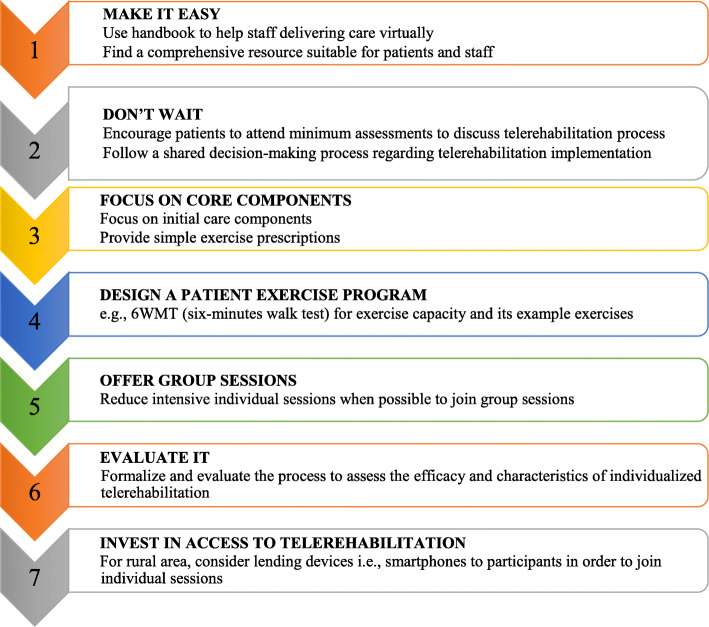
Practical tips for establishing a virtual cardiac rehabilitation program [22]

The transition from conventional rehabilitation into virtual CR needs three main steps. In the first 6 weeks, CR should be done immediately by using available resources. Then in the next 6 months, the delivery should be standardized and optimized. The next step is the delivery should be developed and more innovative. The practical tips for establishing virtual CR can be seen in Fig. 1.

**Fig. 2:**
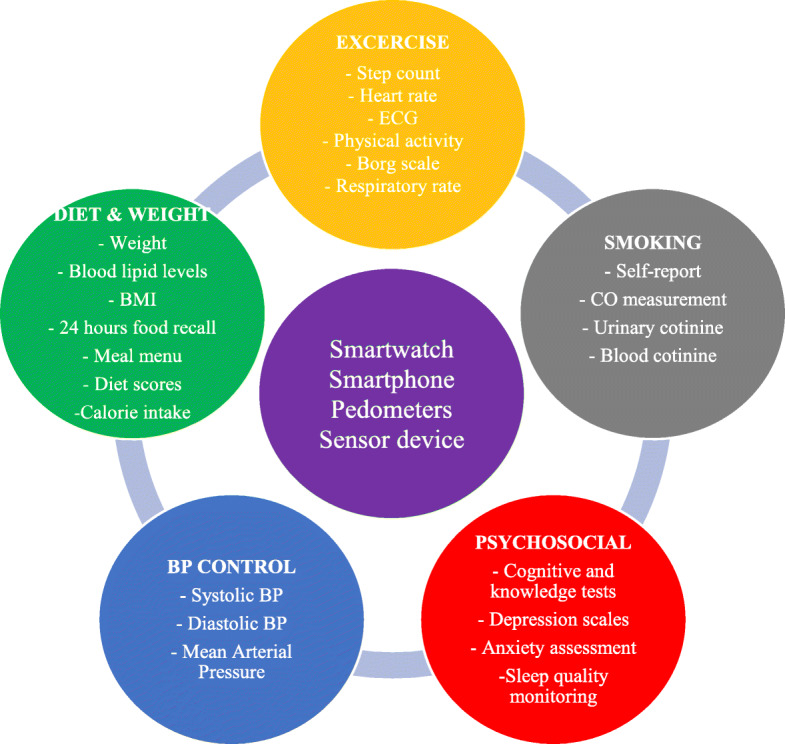
Monitoring devices and parameters of telerehabilitation [23]. BP, blood pressure; CO, carbon monoxide; BMI, body mass index; ECG, electrocardiogram

Access to health care professionals through a portal web of affiliated institutions may reduce costs and promote adherence to sustained healthy lifestyle changes. Interventions using mobile health had been proven effective for improving exercise outcomes in patients with cardiovascular diseases, controlling body weight and body mass index, and improving adherence to medical therapies. The HBCR using mobile health increased participation rates and compliance of participants. Text messaging and the internet are useful for increasing levels of physical activity. A short text message reminding exercises can be applied to increase the number of sessions attended. Figure 2 describes some devices and parameters used in telerehabilitation and monitors the patients [18, 23].

Telerehabilitation appears to be at least as effective as conventional rehabilitation. This delivery model has been successfully trialed in patients with various cardiopulmonary diseases. In a study where HBCR was delivered via computer/tablet, all participants with COPD remained actively participating in the program after 1 year [24].

## Cardiac rehabilitation in pandemic situation

The coronavirus disease-2019 (COVID-19) pandemic has a significant impact on health care globally, including acute and chronic cardiac care. The consequence is the global physical activity levels that dropped dramatically due to policies and the warning which encourage people to live and work at home and keep up physical distancing. Unfortunately, these recommendations do not emphasize the importance of maintaining a healthy lifestyle while staying at home [25, 26].

Recent guidelines from the Centers for Disease Control and Prevention (CDC) recommend all high-risk individuals, including those with cardiovascular risk factors, stay at home to limit the potential exposure to COVID-19. Almost all hospital-based rehabilitation programs are closed, and many elective and outpatient health care activities are canceled or suspended during the COVID-19 pandemic. A consensus guideline from experts recommends limiting direct contact between rehabilitation therapists and patients due to the high transmission rates of COVID-19. The guideline suggests telemedicine as an option for screening and, if possible, for providing treatment to patients. Telemedicine may be considered as electronic personal protective equipment (PPE) by reducing the risk of exposure and contamination for both patients and practitioners [27].

Telemedicine in Indonesia has already been applied and regulated by *Permenkes No. 20 Tahun 2019*. On the other side, the COVID-19 pandemic has led to a large-scale shift toward telemedicine (or virtual health services) in the general population to deliver health care services while still implementing COVID-19 health protocols. It applies to rehabilitation programs that demand sustainability; therefore, new interventions are required to provide rehabilitation services, primarily through telerehabilitation [28].

A study suggests that in the shortened CR program, exercises are focused on the main core components (i.e., education, medical therapy, lifestyle risk management, and psychosocial support) with an individualized approach based on residual cardiac risks, psychological symptoms, and lifestyle assessment. Face-to-face sessions can be replaced with remote assessment and monitoring/guiding through telerehabilitation. Patient assessment and risk stratification are generated by exercise tests whenever possible. Otherwise, other tools can assess cardiovascular risk and physical fitness to provide personalized exercise advice and guide telerehabilitation. For CVD patients who are positive for COVID-19, the exercise program may be postponed if COVID-19’s signs and symptoms are still present. Evaluate exercise resumption is on an individual basis. In general, patients with mild-to-moderate symptoms can gradually restart the exercise program after no fever in a week and no symptoms in 48 h. Whenever possible, all components of the CR program are not suspended but provided remotely [26].

Fifty-two percent of rehabilitation centers in Belgium already provided cardiac rehabilitation programs by implementing telerehabilitation [29]. The remote CR application involved physicians, nurses, dieticians, physiotherapists, and psychologists [29, 30]. All of the CR components were conducted, such as exercise training, dietary advice, smoking cessation guidance, cardiovascular education, psychosocial support, medication adherence, and weight management [13, 29]. The frequency of supervision was diverse, between once a month into several times a week [14, 29].

While at least 35 centers reported delivering CR programs using telerehabilitation in Canada. The remote program reported were using telerehabilitation to deliver exercise program (32% of the programs) and education program (43.5%). Exercise was delivered using telephone, e-mail, mailing forms/resources by post, and web-based platform resources. These methods were significantly increased [6].

The other differences in conducting the CR program during COVID-19 were that the eligibility criteria have shifted to lower risk and less complex patients. The services also are limited, such as initial assessments, consultation, stress management, and group-based education for nutrition [6].

Things that need to be considered in carrying out telerehabilitation are the right staff, the ability to use technology, and other methods of implementing telerehabilitation if the patient does not have access to technology. Developing strategies to admit high-risk/vulnerable populations into the CR program should be concerned [6]. The criteria of a patient in a telerehabilitation program will be challenging during the pandemic. Telerehabilitation implementation needs to prepare technology that ensures communication between teams because CR requires multidisciplinary collaboration [30].

During lockdown, telemedicine can provide many benefits, so it can be a useful intervention to avoid stress and boost the immune system by applying the exercises recommended by CR. It has a real impact on elderly patients with CVD [31].

Patients can still exercise without any equipment during pandemics, such as gymnastic movements of muscular strengthening (squat, sit-to-stand, push-ups against a wall, 1-L water bottles for weights to exercise the upper body, etc.), balance or stretching training, and online relaxation sessions. These exercises can be easily described and explained to the patient by using videos. Moreover, it can be illustrated in a physical activity notebook [32].

An important note that the particular HBCR exercise prescription (more than 30 min of moderate-intensity exercise and 3-5 sessions per week) should have to be modified for individuals who used to walk/run outside. Other forms of physical activity, such as chair-based exercises, calisthenics, resistance, and balance exercises, should be explored. The yoga trends in CR could be a prospect to be used among patients with CVD [33].

By using a logbook, patients can follow the practice of regular training during quarantine. Fifteen minutes per day, every day may be enough to fight against muscle deconditioning and avoid the harmful impacts of strict confinement. However, a precise individualized prescription for each patient is still needed to assure their safety. To increase adherence, coaching by phone seems to be potential and reduces harmful impacts on psychological and physical health, especially in the elderly population [32].

To ensuring patient safety, trackers may be used to quantify physical activity. The equipment is expected to record and send variables (energy expenditure, body mass, glycemia, BP, HR, ECG, etc.) measured via sensors to a web platform accessible to the physician, nurses, and the centers. Simultaneously, real-time monitoring, such as ECG and BP measurement during exercise, is still a challenge to be solved [32].

## Conclusions

The cardiac rehabilitation (CR) program is a sustainable and multidisciplinary intervention where patients are encouraged to continuously implement a healthy lifestyle and regular exercise after starting the program. This program should include physical training, risk factor modification, education, stress management, and psychological support. The CR programs are available in two forms: center-based cardiac rehabilitation (CBCR) and home-based cardiac rehabilitation (HBCR).

The progress made after completing the CBCR program is often not well-maintained for many reasons; therefore, HBCR is highly recommended since patients’ training and participation can be done independently at home. Moreover, long-term adherence to medication and a healthy lifestyle is crucial since they are associated with a lower risk of death and recurrence.

Using multiple technology modalities may be superior to using a single modality. Implementing information and communications technology in medicine, known as telemedicine, maybe a worth alternative to CR. There were already some countries that have been applied telerehabilitation. Moreover, it is highly recommended in the current COVID-19 pandemic, where patients are advised to perform remote consultations unless in urgent conditions. Thus, physicians play an essential role in motivating patients and encouraging their family members to commit to a sustainable CR program with telerehabilitation to facilitate its implementation. It is essential for rehabilitation physicians and family doctors to regularly monitor patients in primary health care facilities.

## Data Availability

Not applicable.

## Abbreviations

AHA: American Heart Association
AICD: Automatic implantable cardioverter-defibrillator
BMI: Body mass index
BP: Blood pressure
CABG: Coronary artery bypass graft
CAD: Coronary artery disease
CBCR: Center-based cardiac rehabilitation
CDC: Centers for Disease Control and Prevention
CHF: Congestive heart failure
CO: Carbon monoxide
COPD: Chronic obstructive pulmonary disease
COVID-19: Coronavirus disease-2019
CR: Cardiac rehabilitation
CVD: Cardiovascular disease
ECG: Electrocardiogram
ESC: European Society of Cardiology
HBCR: Home-based cardiac rehabilitation
HF: Heart failure
HR: Heart rate
HRQoL: Health-related quality of life
LVEF: Left ventricular ejection fraction
NYHA: New York Heart Association
METs: Metabolic equivalent tasks
MI: Myocardial infarction
PCI: Percutaneous coronary intervention
PPE: Personal protective equipment
RPE: Rating of perceived exertion
